# Inferring dynamic gene regulatory networks in cardiac differentiation through the integration of multi-dimensional data

**DOI:** 10.1186/s12859-015-0460-0

**Published:** 2015-03-07

**Authors:** Wuming Gong, Naoko Koyano-Nakagawa, Tongbin Li, Daniel J Garry

**Affiliations:** 1 Lillehei Heart Institute, University of Minnesota, 2231 6th St S.E, 4-165 CCRB, Minneapolis, MN 55114 USA; 2 AccuraScience LLC, 5721 Merle Hay Road, Suite #16B, Johnston, IA 50131 USA

**Keywords:** Cardiac differentiation, Network inference, Logistic regression, Time-varying dynamic Bayesian model, Data integration, Gene regulatory network

## Abstract

**Background:**

Decoding the temporal control of gene expression patterns is key to the understanding of the complex mechanisms that govern developmental decisions during heart development. High-throughput methods have been employed to systematically study the dynamic and coordinated nature of cardiac differentiation at the global level with multiple dimensions. Therefore, there is a pressing need to develop a systems approach to integrate these data from individual studies and infer the dynamic regulatory networks in an unbiased fashion.

**Results:**

We developed a two-step strategy to integrate data from (1) temporal RNA-seq, (2) temporal histone modification ChIP-seq, (3) transcription factor (TF) ChIP-seq and (4) gene perturbation experiments to reconstruct the dynamic network during heart development. First, we trained a logistic regression model to predict the probability (LR score) of any base being bound by 543 TFs with known positional weight matrices. Second, four dimensions of data were combined using a time-varying dynamic Bayesian network model to infer the dynamic networks at four developmental stages in the mouse [mouse embryonic stem cells (ESCs), mesoderm (MES), cardiac progenitors (CP) and cardiomyocytes (CM)]. Our method not only infers the time-varying networks between different stages of heart development, but it also identifies the TF binding sites associated with promoter or enhancers of downstream genes.

The LR scores of experimentally verified ESCs and heart enhancers were significantly higher than random regions (p <10^−100^), suggesting that a high LR score is a reliable indicator for functional TF binding sites. Our network inference model identified a region with an elevated LR score approximately −9400 bp upstream of the transcriptional start site of *Nkx2-5*, which overlapped with a previously reported enhancer region (−9435 to −8922 bp). TFs such as Tead1, Gata4, Msx2, and Tgif1 were predicted to bind to this region and participate in the regulation of *Nkx2-5* gene expression. Our model also predicted the key regulatory networks for the ESC-MES, MES-CP and CP-CM transitions.

**Conclusion:**

We report a novel method to systematically integrate multi-dimensional -omics data and reconstruct the gene regulatory networks. This method will allow one to rapidly determine the cis-modules that regulate key genes during cardiac differentiation.

**Electronic supplementary material:**

The online version of this article (doi:10.1186/s12859-015-0460-0) contains supplementary material, which is available to authorized users.

## Background

Decoding the temporal control of gene expression patterns is essential to understand the complex mechanism of developmental regulatory events during heart development. High-throughput methods have been employed to systematically study the dynamic and coordinated nature of cardiac differentiation at the global level with multiple dimensions [1-6]. For example, in several studies, RNA-seq and histone modification ChIP-seq experiments were performed to profile the changes in global gene expression and the chromatin state at distinct stages of cardiac differentiation from ESCs to cardiomyocytes in human and mouse [1,3]. In these reports, the authors reported changes in chromatin modification patterns associated with gene activation and identified stage specific distal enhancer elements. He et al., outlined the candidate binding sites of five known cardiac transcription factors (TFs) (Gata4, Nkx2-5, Tbx5, Srf and Mef2a), which were identified using ChIP-seq [2]. Moreover, Schlesinger et al. knocked down each of the four key cardiac transcription factors (Gata4, Mef2a, Nkx2-5 and Srf) in HL-1 cells using RNA interference, followed by the profiling of the changes in global gene expression [4]. Although these studies presented a novel and global perspective for the examination of the chromatin status and the prediction of transcriptional regulation, they were limited in the types of data that were integrated [1,3] and they based their initial screening on a small set of candidate TFs [2,4]. As large-scale multi-dimensional data are accumulating at an unprecedented pace, there is a pressing need to develop systematic methods to integrate these data from individual studies and infer the dynamic gene regulatory networks (GRN) during cardiac differentiation in an unbiased manner.

Time series expression profiles based on microarray and/or more recently RNA-seq data have been widely used to reconstruct the static networks, that is, networks with invariant topology over a given set of genes [7-11]. However, because the GRN at a particular time point depends on a specific biological context, it can undergo systematic rewiring rather than being invariant over time. Therefore, recent research has focused on inferring the dynamic (time-varying) networks over the time course [1-4,12-15]. A key technical hurdle to precisely reconstruct dynamic networks based solely on temporal expression data is that there are too many unknown variables to be estimated (i.e. *(T-1)p* ^2^ network edges). Some attempts have been made to circumvent this difficulty including: factorizing gene-gene regulatory relationships into modular effects [1,3,11,14], deconvolving the observed indirect effects into direct effects [2,16], or smoothing the edge weight between the networks of neighboring time points [4,13,17]. However, the overall performance of reconstructing GRN based solely on temporal expression profiles is still limited [1,3,18].

One widely used strategy to infer the causal relationship in GRN is to over-express or repress the key TFs and measure the change in global expression. The significantly up- or down-regulated genes may be either directly or indirectly regulated by the perturbed TFs. This strategy has been successfully utilized and several examples include: the GRN in sea urchin embryonic development [2,4,19], the early response of GRN in embryonic stem cells (ESC) [7-11,20], and the cardiac GRN involving several key cardiac genes [4]. Perturbation-based methods can, in theory, greatly improve the prediction accuracy for downstream targets, as compared with the methods solely based on temporal expression profiles [18]. The limitation of this strategy is that it is unrealistic to perturb all TFs in the mammalian genome in a specific context and it is not easy to distinguish direct effects from indirect effects in the readout.

The most common strategy used to discover the direct regulatory relationship is to combine the TF information and temporal expression profiles [2,12,21-23]. The general assumption is that a gene can be regulated by a TF if its promoter or enhancer regions are occupied by the TF. The TF binding sites (TFBS) within the putative regulatory region of a gene are identified by either scanning the known positional weight matrix (PWM) representing a relatively short (5–20 nucleotides) degenerative sequence motif recognized by a TF, or by TF ChIP-seq experiments. Although PWMs have been defined for the TFBSs of more than 500 TFs in vertebrates by various techniques [24-32], the sensitivity and specificity are generally low when used to predict putative binding sites [33]. Alternatively, TF ChIP followed by sequencing or microarray analyses emerged as the standard approach to directly determine the *bona fide* TFBS. However, because ChIP-seq experiments are still relatively expensive and labor-intensive, and the TFBSs tend to vary in distinct biological contexts, for example, only 7.14% of enhancers identified in ESCs are overlapped with the enhancers in heart [34-37], the number of available TF ChIP-seq datasets is still limited. Moreover, for most TFs in the genome, there are no ChIP-seq datasets available. For example, in ChIPBase, only 12 and 5 TFs have corresponding ChIP-seq data in ESCs and cardiomyocyte HL-1 cells, respectively [38]. At present, there is no consensus regarding whether ChIP-seq data obtained in one cell type can be readily applied to predict TFBS in another cell type. Moreover, it is unclear whether or not we can adapt the information from the available ChIP-seq results and predict the binding sites of TFs with only PWM information in a specific biological context (e.g. cell types or developmental stages).

In lieu of profiling the binding sites of individual TFs, the general enhancers or regulatory regions have also been mapped by DNaseI hypersensitive sequencing experiments as well as ChIP-seq with p300, histone H3 Lys4 mono-methylation (H3K4me1), histone H3 Lys27 acetylation (H3K27ac) in a wide range of cell types [39-44] including mouse ESCs and the heart [34-36,45]. The genomic loci defined by these marks, however, typically span several hundred or thousand bases, and are generally too broad to define the specific DNA sequences mediating promoter or enhancer functions. It has been proposed that local depletion in the ChIP signal intensity (dip) is indicative of TF binding sites [41]. Thus, several studies have used the structural change of these active marks to discover the functional TFBS among the enhancer regions, either by heuristic methods [1], or by more sophisticated approaches, such as an integrated hidden Markov model [46], logistic regression [47,48], or a hierarchical mixture model [49]. However, these studies usually focused on individual cell types. Moreover, they focus on static regulatory relations and do not fall under the framework of inferring dynamic gene regulatory networks.

While each of the aforementioned strategies has its own merits, they also have limitations in the inability to capture the dynamic networks. An integrated approach for network inference, which combines the strengths of all these methods is highly desirable. In this study, we presented a framework to integrate available four-dimensional data: (1) temporal RNA-seq, (2) temporal histone ChIP-seq, (3) TF ChIP-seq and (4) perturbation studies to reconstruct the dynamic networks during cardiac differentiation. Our method not only infers the time-varying networks between distinct stages of heart development, but also identifies the TF binding sites on the promoter or enhancer of the genes being regulated.

## Results

### Overview

**Figure 1:**
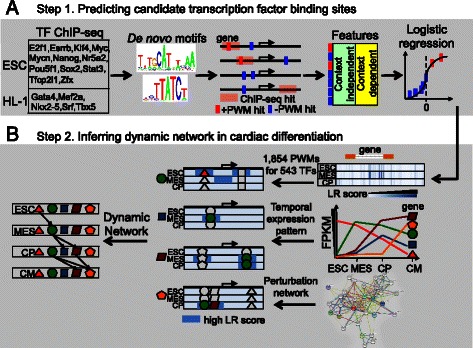
**Overview of our two-step strategy to infer dynamic regulatory networks during cardiac differentiation. (A)** Training a general logistic regression model to predict the probability being bound by any transcription factor (LR score) in 40 kb cis-regions surrounding transcriptional start sites (TSS) of expressed genes. The response variables of the model indicate whether the hit of the PWM of a specific TF coincides with the peak region of the corresponding ChIP-seq data. 15 context-independent (e.g. conservation) and 54 context-dependent (e.g. mean intensity of H3K27ac in 1 kb surrounding any base) features were used to train the logistic regression model. **(B)** Context-dependent LR score, temporal expression profiles and perturbation networks were used to infer the dynamic regulatory networks based on a time-varying dynamic Bayesian network model.

We developed a two-step strategy to infer the dynamic GRN during cardiac differentiation (Figure 1). In the first step, based on 17 TFs whose ChIP-seq data are available for either mouse ESCs or cardiomyocyte HL-1 cells (Table 1), we trained a logistic regression model to predict the probability for any base being bound by any TFs with known PWMs, at a specific differentiation stage. The model included the context independent features that do not change during differentiation (e.g. base conservation) and context dependent features such as the expression levels of nearby genes, the intensity of histone modifications within defined distances, as well as histone modification changes between adjacent time points. This concept was modified from the work by Ernst et al. that infers a score quantifying the general binding preferences of TFBS [48]. However, it should be noted that, for any given sequence in the genome, the output of our model, the logistic regression (LR) score, was dependent upon the differentiation stage, and not specific TFs. Specifically, the stage-specific LR score was designed to capture the stage-specific TFBS. In the second step, we used the following information: (1) the temporal LR score defined in step 1, (2) the temporal expression profiles of cardiac differentiation at four developmental stages in the mouse [ESCs, mesoderm (MES), cardiac progenitors (CP), and cardiomyocytes (CM) [1]], and (3) the perturbed network we compiled from the perturbation experiments performed in mouse ESCs and HL-1 cells [2,4,20,50,51], which were combined under the framework of a time-varying dynamic Bayesian network model to infer the dynamic networks during cardiac differentiation.

**Table 1:** **Transcription factor ChIP-seq datasets used to train the logistic regression model**

**Regulator**	**Cell type**	**Positional weight matrix**	**Source**
E2f1	ESC		[52]
Esrrb	ESC		[26]
Gata4	HL-1		[2]
Klf4	ESC		[26]
Mef2a	HL-1		[2]
Myc	ESC		[26]
Mycn	ESC		[26]
Nanog	ESC		[26]
Nkx2-5	HL-1		[2]
Nr5a2	ESC		[74]
Pou5f1	ESC		[26]
Sox2	ESC		[26]
Srf	HL-1		[2]
Stat3	ESC		[26]
Tbx5	HL-1		[2]
Tfcp2l1	ESC		[26]
Zfx	ESC		[26]

### Stage specific transcription factor binding probability (LR score)

First, we compiled the ChIP-seq data for 12 TFs (E2f1, Esrrb, Klf4, Myc, Mycn, Nanog, Nr5a2, Pou5f1, Sox2, Stat3, Tfcp2l1 and Zfx) in mouse ESCs and 5 TFs (Gata4, Mef2a, Nkx2-5, Srf and Tbx5) in HL-1 cells from ChIPBase. The PWMs for each TF were inferred by using HOMER (Table 1) [38,52]. The 17 *de novo* PWMs derived from these ChIP-seq experiments were used to scan the 40 kb cis-region surrounding the transcriptional start site (TSS) of the 13,961 genes whose expression levels were greater than 1 FPKM in at least one of the four time points: ESCs, MES, CPs and CMs in the RNA-seq experiments described in Wamstad et al. [1] (see Methods). Although the PWMs were derived from the corresponding ChIP-seq dataset, due to their degenerative nature, we still expected to obtain PWM hits that did not overlap with the ChIP-seq peaks. If a PWM hit overlapped with the ChIP-seq peak of the corresponding TF, the center base of the PWM was considered to be a positive response variable in the logistic regression model, otherwise, it was considered a negative response variable. For any given base in the cis-region, the features of the logistic regression model included 15 sequence-based context independent features (Table 2), 4 features regarding the expression levels of the nearby genes at time *t* and *t + 1* (Table 2), 50 features based on the intensity of four histone marks (H3K27ac, H3K4me1, H3K4me3, H3K27me3) and RNA polymerase II phosphorylation at serine 5 (RNAP) profiled in Wamstad et al. [1] (Table 3). The 15 sequence-based features (features #1 - #15) were defined as described in Ernst et al. [48]. As the genuine TF binding sites were expected to lead to alterations in the expression levels of the nearby genes, we included expression levels of the nearby genes as the features for modeling training (features #16 - # 19). For the 12 TFs included in the model for the ESCs, time *t* was defined as the ESC stage, and *t + 1*, the MES stage, while for 5 cardiac TFs, time *t* was defined as the CP stage and *t + 1* was the CM stage. To capture the structural changes of histone modifications during cardiac differentiation, for each histone mark (including DNaseI hypersensitive signals and RNA Pol II signals), we defined five features for the mean intensity within the surrounding window of different sizes (10, 50, 100, 500 and 1000 nt), as well as their changes from time *t* to *t + 1* (features #20 - #69).

**Table 2:** **The 19 features based on sequence and nearby gene expression**

**No.**	**Description**	**Dir.**	**P value**	**Signif.**
1	PhastCons score for 60-way vertebrate alignments; 0 if not available	+	1.82E-09	***
2	PhastCons score for placental mammal; 0 if not available	+	7.76E-31	***
3	1 if PhastCons vertebrate score is available and the score is 0; 0 otherwise	+	1.55E-02	*
4	1 if PhastCons placental score is available and the score is 0; 0 otherwise	-	4.53E-04	***
5	1 if PhastCons vertebrate score is available; 0 otherwise	+	7.24E-125	***
6	1 if PhastCons placental score is available; 0 otherwise	+	8.36E-01	
7	1 if base is in CpG islands; 0 otherwise	+	1.03E-23	***
8	ln(x + 5), where x is the absolute number of base pairs to nearest RefSeq transcription start site	+	1.69E-02	*
9	1 if base is part of repeat element based on RepeatMaster; 0 otherwise	-	6.07E-02	
10	1 if base is part of a transcribed region of a RefSeq gene; 0 otherwise	-	2.76E-07	***
11	1 if base is between the start and end of the coding region of the gene; 0 otherwise	-	3.15E-05	***
12	1 if base is part of RefSeq exon; 0 otherwise	+	7.47E-03	**
13	1 if base is part of a RefSeq exon and within the coding region of the gene; 0 otherwise	-	2.07E-105	***
14	1 if base is part of a RefSeq intron; 0 otherwise	+	2.19E-02	*
15	Percentage of G or C base pairs of all bases within 50 bases in either direction	+	0.00E + 00	***
16	ln(x + 1), where x is the FPKM of nearest gene at time t	-	1.20E-07	***
17	ln(x + 1), where x is the FPKM of nearest gene at time t + 1	+	3.81E-05	***
18	1 if nearest gene is significantly up-regulated from t to t + 1; 0 otherwise	+	2.05E-03	**
19	1 if nearest gene is significantly down-regulated from t to t + 1; 0 otherwise	+	5.77E-02	

*: 0.01≤*p* value < 0.05; **: 0.001≤*p* value < 0.01; ***: *p* value < 0.001.

**Table 3:** **The 50 features based on ChIP-seq intensity of four histone marks (H3K27ac, H3K4me1, H3K4me3 and H3K27me3) and RNA polymerase II phosphorylation at serine 5 (RNAP)**

**No.**	**Histone**	**Description**	**Dir.**	**P value**	**Signif.**
20	H3K27ac	mean(x_t_,1000)	+	4.40E-245	***
21		mean(x_t_,500)	-	7.63E-03	**
22		mean(x_t_,100)	-	1.59E-21	***
23		mean(x_t_,50)	+	2.41E-06	***
24		mean(x_t_,10)	-	2.09E-01	
25		mean(x_t+1_,1000)-mean(x_t_,1000)	-	1.62E-04	***
26		mean(x_t+1_,500)-mean(x_t_,500)	-	9.25E-01	
27		mean(x_t+1_,100)-mean(x_t_,100)	-	1.03E-03	**
28		mean(x_t+1_,50)-mean(x_t_,50)	+	6.40E-04	***
29		mean(x_t+1_,10)-mean(x_t_,10)	-	1.33E-01	
30	H3K4me1	mean(x_t_,1000)	+	9.27E-01	
31		mean(x_t_,500)	+	2.04E-07	***
32		mean(x_t_,100)	+	1.04E-01	
33		mean(x_t_,50)	-	8.89E-01	
34		mean(x_t_,10)	-	3.06E-01	
35		mean(x_t+1_,1000)-mean(x_t_,1000)	-	1.21E-56	***
36		mean(x_t+1_,500)-mean(x_t_,500)	+	2.42E-17	***
37		mean(x_t+1_,100)-mean(x_t_,100)	+	4.97E-05	***
38		mean(x_t+1_,50)-mean(x_t_,50)	-	4.04E-01	
39		mean(x_t+1_,10)-mean(x_t_,10)	+	8.39E-01	
40	H3K4me3	mean(x_t_,1000)	-	3.34E-91	***
41		mean(x_t_,500)	+	2.34E-32	***
42		mean(x_t_,100)	+	1.28E-02	*
43		mean(x_t_,50)	-	8.41E-01	
44		mean(x_t_,10)	+	7.33E-01	
45		mean(x_t+1_,1000)-mean(x_t_,1000)	-	3.73E-23	***
46		mean(x_t+1_,500)-mean(x_t_,500)	+	3.82E-01	
47		mean(x_t+1_,100)-mean(x_t_,100)	+	3.15E-04	***
48		mean(x_t+1_,50)-mean(x_t_,50)	+	9.68E-01	
49		mean(x_t+1_,10)-mean(x_t_,10)	-	7.65E-01	
50	H3K27me3	mean(x_t_,1000)	+	1.61E-15	***
51		mean(x_t_,500)	-	1.83E-27	***
52		mean(x_t_,100)	+	7.01E-02	
53		mean(x_t_,50)	-	2.04E-01	
54		mean(x_t_,10)	-	8.98E-02	
55		mean(x_t+1_,1000)-mean(x_t_,1000)	+	1.77E-08	***
56		mean(x_t+1_,500)-mean(x_t_,500)	-	9.39E-05	***
57		mean(x_t+1_,100)-mean(x_t_,100)	+	2.15E-01	
58		mean(x_t+1_,50)-mean(x_t_,50)	-	3.05E-01	
59		mean(x_t+1_,10)-mean(x_t_,10)	-	4.10E-01	
60	RNAP	mean(x_t_,1000)	-	0.00E + 00	***
61		mean(x_t_,500)	+	1.82E-105	***
62		mean(x_t_,100)	+	1.04E-13	***
63		mean(x_t_,50)	-	1.54E-01	
64		mean(x_t_,10)	+	9.46E-01	
65		mean(x_t+1_,1000)-mean(x_t_,1000)	-	6.44E-166	***
66		mean(x_t+1_,500)-mean(x_t_,500)	+	1.98E-31	***
67		mean(x_t+1_,100)-mean(x_t_,100)	+	5.17E-03	**
68		mean(x_t+1_,50)-mean(x_t_,50)	-	4.77E-01	
69		mean(x_t+1_,10)-mean(x_t_,10)	+	1.96E-01	

*: 0.01 ≤*p* value < 0.05; **: 0.001≤*p* value < 0.01; ***: *p* value < 0.001.

**Figure 2:**
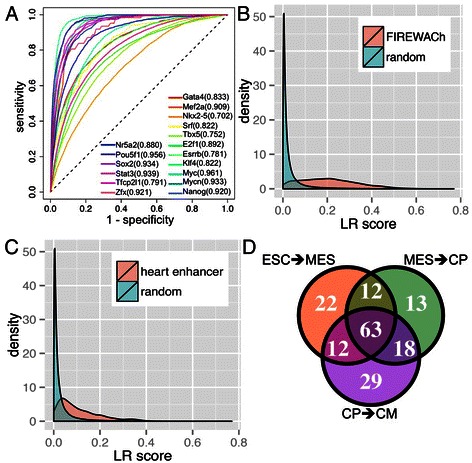
**Stage specific transcription factor (TF) binding probability (LR score). (A)** Performance of leave-one-TF-out cross-validation of predicting binding sites of 12 ESC TFs and 5 cardiac TFs, as measured by area under the Receiver Operating Characteristics curve (AUC). **(B)** Distribution of the mean LR score of ESC transcriptional regulatory modules identified by functional identification of regulatory elements within accessible chromatin (FIREWACh) and the LR score of one million randomly selected bases in the cis-region [35]. **(C)** Distribution of the mean LR score of heart enhancers and the LR score of one million randomly selected bases in the respective cis-regions [34]. **(D)** Number of significantly enriched TFs in high LR score regions (>0.1) in the three stage transition.

As our goal was to train a general stage-specific model to predict the binding probability of any TF with PWM, we used a leave-one-TF-out cross-validation (LOTFOCV) to evaluate the generalizability of the model. In short, at each stage (time point), we used the data from 16 of the 17 TFs to train a model, and tested its performance on the remaining TFs. The sensitivity and specificity of the predictions were determined by the overlap between the PWM hit and the ChIP-seq peaks. The performance was measured by Area Under Receiver Operating Characteristics Curve (AUC) (Figure 2A). The AUC ranged from 0.961 (Mycn) to 0.702 (Nkx2-5) with the 40 kb cis-region and PWM score cutoff at 90%, while the mean AUC of 17 TFs was 0.860 (Figure 2A). We also checked the AUC with distinct parameters (cis-region = 20 kb or 40 kb, PWM score cutoff = 90% or 95%), and noted that the performance was similar between these conditions (Additional file 1: Figure S1A-C). We compared the performance of the full model by using all features (features #1 - #69), models without features defined at *t + 1* (features #1 - #15, #16, #20 - #24, #30 - #34, #40 - #44, #50 - #54, #60 - #64), and models with only sequence features (features #1 - #15) (Additional file 1: Figure S1D). The full model demonstrated the best performance for 14 out of 17 TFs, with the exception of Myc, Nkx2-5 and Tbx5. The results suggested that the model was able to predict the stage-specific TFBSs by using context independent sequence features combined with context dependent expression and histone modification features. Because the features *per se* were independent of the PWMs of any specific TF, this model can be used to predict the binding probability of other TFs, whose ChIP-seq data are not available during the cardiac differentiation process yet the PWMs of which have already been defined.

Next, we trained the logistic regression model using the data from all 17 TFs. We found that sequence features such as conservation (#2) and GC content (#15) had significant positive effects on the binding probability (LR score) (Wald test *p* value = 7.76E-31 and <1E-100), and being on a coding sequence (CDS) (#13) had significant negative effects on the LR score (*p* value < 1E-100), which was predicted for transcription factors (Table 2). As for histone modification and RNA Pol II features, high H3K27ac in the 1 kb surrounding region (#20) had significantly positive effects on the LR score (*p* value < 1E-100), while high H3K4me3 and RNA Pol II in the 1 kb surrounding region (#40 and #60) had significantly negative effects (*p* value = 3.34E-91 and < 1E-100). The model also successfully captured the local dip of histone marks as the H3K27ac in the 100 bp surrounding region had negative effects on the LR score (*p* value = 1.59E-21), consistent with previous reports [41]. It is interesting to note that the changes of H3K4me1, H3K4me3 and RNAP from t to t + 1 (#35, #45 and #65) also demonstrated significantly negative effects on the LR scores (p value = 1.21E-56, 3.73E-23 and <1E-100), suggesting that reduced levels of these three histone modification marks may be a novel signature of TF binding sites.

It should be noted that we trained the logistic regression model without any regularization. To demonstrate the robustness of the obtained feature coefficients under the regularization, we used elastic net regularized logistic regression to fit the input data from all 17 TFs (see Methods). We found that the correlation coefficients between the feature coefficients estimated by the logistic regression model without regularization and with LASSO regularization is 0.853 (Additional file 1: Figure S3A). The correlation coefficient between the predicted LR score is greater than 0.99 for all cis-regions on mouse chromosome 10 (Additional file 1: Figure S3B, C and D). These results suggested that not only are the estimated coefficients robust using the LASSO regularization, but the predicted LR scores are also highly consistent.

To examine whether the high LR score is indicative of a functional TFBS, we compared the LR scores of known ESCs and heart enhancers with those of cis-regions of randomly chosen genes. We determined that the mean LR scores on known enhancers from four independent studies were all significantly higher than cis-regions of the randomly chosen genes (Wilcoxon rank-sum test *p* value < 1E-100, Figure 2A, 2B, Additional file 1: Figure S2A, B) [34-36,45]. It should be noted that the difference was particularly strong on the recently defined ESC transcriptional regulatory modules that have a relatively short range (mean width = 185.8 bp) [35], compared to the other three studies (mean width = 686.7, 2389.3 and 19177 bp, respectively). The results suggest that a high LR score can be used as a valid indicator for functional TFBS.

As outlined above, the base-wise LR score was stage-specific. We decided to investigate the significantly enriched PWMs of TFs in high LR score regions (>0.1) in each stage transition (from ESCs to MES, from MES to CPs and from CPs to CMs). We compiled a large number of (1,854) PWMs for 543 TFs from multiple sources [24-32]. 1,236 PWMs from 362 TFs expressed (FPKM > 1) in ESCs, MES or CPs were used in the following analysis (Additional file 1: Figure S4A). If one TF had multiple PWMs, the PWM with the lowest *p* value was reported. We found that TFs had distinct enrichment patterns in each stage transition (Figure 2D and Additional file 2: Table S1). We conducted pathway enrichment analysis, which demonstrated that 63 TFs that were significantly enriched in high LR score regions in all three transitions had significantly enriched functions in cell cycle regulation (binomial test *p* value = 4.05E-05) (Additional file 2: Table S1). The 22, 13 and 29 TFs that are specifically enriched in ESC-MES, MES-CP and CP-CM transitions were significantly enriched for stem cell maintenance (*p* value = 9.36E-3), cell fate specification (*p* value = 2.59E-4) and cardiovascular system developmental pathways (*p* value = 2.65E-3), respectively. In summary, we established the LR score as an effective metric to predict the stage-specific binding probability of putative transcription factor binding sites.

### Inferring dynamic regulatory networks during cardiac differentiation

Next, we integrated (1) the stage-specific LR scores, (2) the temporal expression profiles and (3) the perturbation data under a time-varying dynamic Bayesian network (DBN) framework to infer the dynamic regulatory networks during cardiac differentiation. Song et al. have developed a DBN framework to infer the time-varying direct or indirect networks by smoothing the edge changes between adjacent networks under the assumption that adjacent networks are likely to share common edges than temporally distal networks [13,17]. This framework is conceptually flexible and computationally efficient. We extended this framework to model the impact (weights) of a list of TFs to a downstream target as the multiplicity between the weights of overlapping windows in the cis-region surrounding the target and a weighted binding matrix. The weighted binding matrix measures (1) whether or not TF PWM hits exist, (2) the LR score of the window and (3) whether or not the targets have been significantly affected in the perturbation experiments of the TFs (see Methods).

For any gene expressed in the cardiac differentiation process, this model can predict (1) which TFs are the direct regulators, and moreover, (2) the regulatory binding sites on the cis-region (TFBS). The *p*-value of each predicted TFBS was evaluated using a bootstrap method (see Methods). The predicted regulatory relations (links) of 13,961 expressed genes are shown in Additional file 2 Table S2. The fractions of expression variance explained by the model (cis-region = 40 k, PWM score cutoff = 90%) were 88.6%, 88.1% and 88.2%, for MES, CP and CM, respectively.

**Figure 3:**
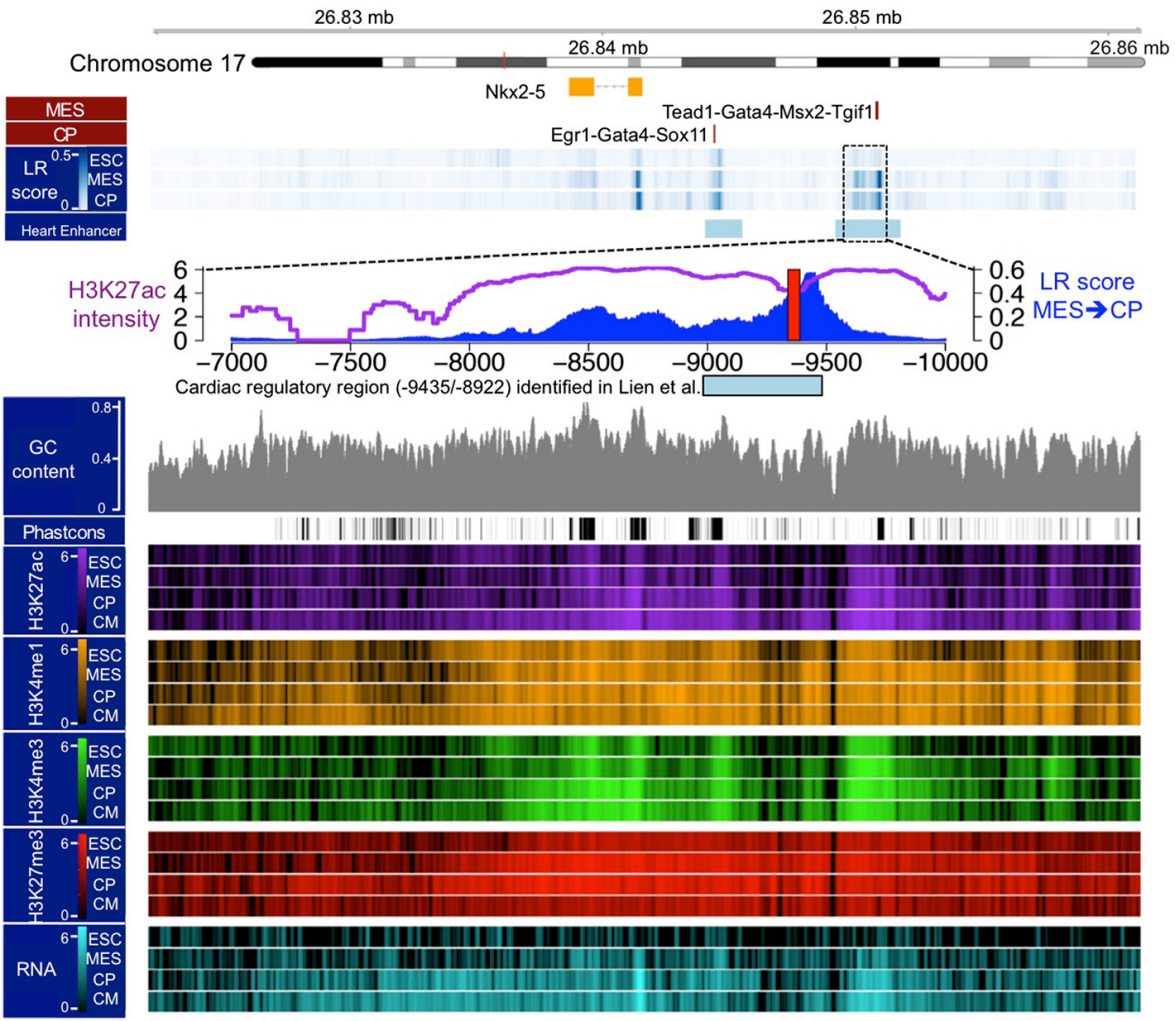
**Predicted transcription factor binding sites around the 40-kb cis-region of the** ***Nkx2-5*** **gene.** The cardiac regulatory region (−9435/-8922) has been reported by Lien et al. Brown bars indicate the presence of links and associated transcription factors at distinct stage transitions.

Nkx2-5 is one of the essential transcription factors mediating heart development. Without Nkx2-5 function, the heart primordium does not loop properly and embryos die at embryonic day (E) 9.5 [53,54]. It has been reported that a region −9435 bp to −8922 bp upstream of *Nkx2-5*'s TSS contains an enhancer that controls its early cardiac-specific transcription and this regulation is Gata-dependent [55,56]. Our network inference model predicted that this region contains a high LR score region and peaks approximately −9400 bp upstream of TSS (Figure 3). Around this peak LR score, there was a dip of H3K27ac that contains the clustered binding sites of the Hippo signaling pathway player Tead1, Gata4, BMP signaling pathway players, Msx2 and Tgif1. Tead1 binding motif is known to be enriched around sequences pulled down by p300, Gata4, Nkx2-5, and Mef2a using ChIP assays [2]. Msx1 and Msx2 functions have been implied in endothelial-mesenchymal transformation of the atrioventricular cushions and patterning of the atrioventricular myocardium. BMP signaling pathway is an important regulator of heart development [57]. Although it is still unknown whether these factors directly bind to the *Nkx2-5* regulatory region, we predict that this regulatory module may be functionally important to activate *Nkx2-5* in cardiac progenitors [58-60]. Additional file 1: Figure S6A-C are additional examples of *Gata4, Gata6, and Bhlh40* genes demonstrating the overlap of predicted TFBS and experimentally detected enhancers. These individual examples demonstrate that our network inference model identified many biologically verified links and suggests that novel links may be of biological significance.

**Figure 4:**
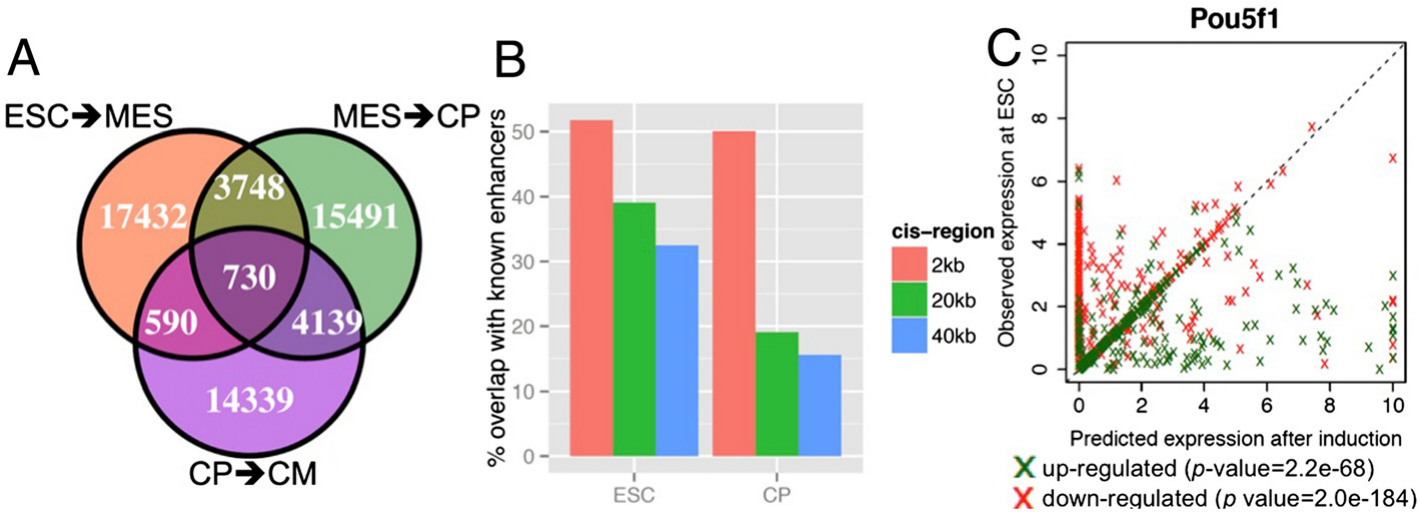
**Inferring dynamic networks during cardiac differentiation by a time-varying dynamic Bayesian network model. (A)** Number of inferred gene-gene regulatory relationships in ESC-MES, MES-CPs, and CPs-CMs transitions. **(B)** Number of predicted transcription factor binding sites that are overlapped with known ESC and heart enhancers on genes that have enhancers within their 2 kb, 10 kb or 40 kb regions surrounding the transcriptional start sites. **(C)** Predicted up- or down-regulated genes on computationally inducing *Pou5f1* five-fold in ESCs compared with known up- or down-regulated genes on experimentally inducing Pou5f1. p-values were determined using Fisher's exact test.

A total of 17,432, 15,491 and 14,339 positive edges (***u*** ^*i,t*^ >0, see Methods) were predicted for the ESC-MES, MES-CP and CP-CM transitions, respectively (Figure 4A). The common links between ESC-MES and MES-CP, and between MES-CP and CP-CM, represented 10.6% and 12.6% of the total number of discovered edges in the corresponding time points. The number of common links between ESC-MES and MES-CP (3,748) and between MES-CP and CP-CM (4,139) were significantly higher than the common ones between ESC-MES and CP-CM (590), suggesting that the common links were captured between adjacent networks.

Overall, 51.7% and 50.0% of predicted TFBSs overlapped with known ESCs and heart enhancers, respectively, on genes that have mapped enhancers within their 2 kb regions surrounding their TSS's [34-36,45] (Figure 3B). In comparison, these ratios decreased for the more distant TFBS (e.g. 39.1% and 15.5% for ESCs and heart enhancers over a 40 kb cis-region). These results suggested that the model was particularly good at capturing the TFBS near the TSS. The superior performance of the ESC model over the CP model may be due to the fact that there were more data used in the training of the ESC model.

Next, to evaluate the predictive power of the model we performed a "computational perturbation experiment". We computationally increased the expression levels of key ESC TF genes (*Pou5f1*, *Tcf3*, *Sox2*, *Nanog* and *Zfp281*) by five-fold in ESCs, and then predicted the global expression profiles after computational 'induction' and compared the results with the published experimental data upon over-expression of these TFs [20]. We found that there was a significant agreement in the direction of response (up- or down-regulation) of gene expression between the computational overexpression and the experimental data, indicating the high predictability of this model (Fisher's exact test p value = 2.2E-68 and <1E-100 for Pou5f1) (Figure 4C and Additional file 1: Figure S5).

**Figure 5:**
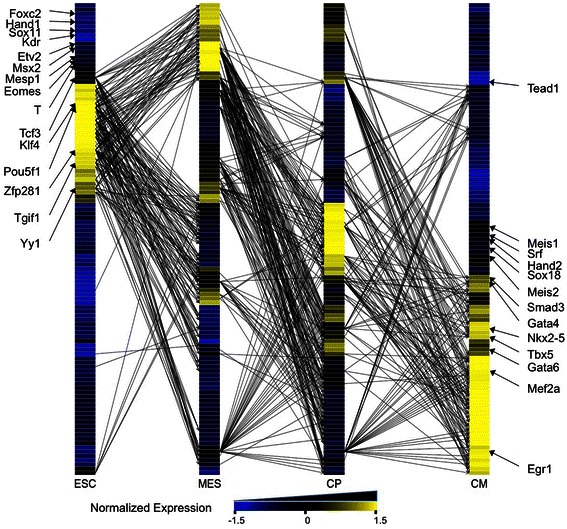
**Inferred dynamic regulatory networks of 94 genes during cardiac differentiation (ESCs-MES, MES-CPs and CPs-CMs).** Expression levels for each gene were normalized to a mean of zero and a standard deviation of one.

Figure 5 is a graphic representation of the positive links predicted in the dynamic regulatory networks involving a selected list of 93 key genes in cardiac differentiation. This representation clearly illustrates the changes of gene expression according to the differentiation states as well as the dynamic gene regulatory network involved in this process. Of note, pluripotency genes such as *Pou5f1*, *Klf4* or *Zfp281* had the greatest number of links to the predicted down-stream targets in ES to MES transition. The targets included early mesodermal genes such as *T*, *Mesp1*, *Eomes*, *Kdr*, as well as early lineage specific regulators, such as *Etv2*, *FoxC2*, *Sox11*, *Sox18* (endothelial), *Nkx2-5*, *Gata4*, *Gata6*, *Hand1*, *Hand2* and *Tbx5* (cardiac), but did not include the cardiac structural genes. Of the identified targets, the early mesodermal genes peaked at the MES stage, however the lineage specific regulators peaked later at the CP stage, although the link was identified in the ES-MES transition. This likely reflects the changes in the histone modification patterns that precede gene activation [1]. In the MES-CP transition, many links from mesodermal genes to cardiac structural genes, as well as from lineage specific genes to cardiac structural genes were identified. In addition, links emanating from several hub genes such as *Msx2*, *Egr1* and *Yy1* were prominent. Although the functions of these factors in cardiac development is not well defined, this result suggests the involvement of these factors in the cardiogeneic process [2,59,61-63]. In the CP-CM transition, Tcf3, Egr1, Nkx2-5, Gata4, Srf, Smad3 and Meis2 are predicted to activate many highly expressed genes in cardiomyocytes. Interestingly, although the same target genes (cardiac structural genes) are activated in the MES-CP and CP-CM transitions, the group of activating genes changed from MES and CP, likely reflecting the changes in transcriptional regulatory machinery.

It should be noted that the time-varying DBN modeling required known PWMs, and for more than 70% of the TFs, their PWMs have not been identified yet. Moreover, non-DNA binding proteins, such as signaling proteins and those associated with the chromatin complex, also play important roles in ESC differentiation and heart development by interacting with or modifying the TFs [64-66]. To incorporate the effects of these proteins into the time-varying DBN, we evaluated an extended model using information of protein-protein interaction (see Additional file 3). This extended model predicted additional gene regulatory pathways (such as SWI/SNF complex) that are important for heart development (Additional file 1: Figure S7).

## Discussion

Owing to the rapid declining cost of sequencing experiments and our deep understanding of the roles of histone modification during transcriptional regulation, the temporal RNA-seq and histone ChIP-seq data are emerging as powerful tools to explore the biological dynamics, especially during the developmental process [1,3,67,68]. In this study, we propose a novel method to integrate such multi-dimensional data to predict transcription factor binding sites and gene regulatory networks. Instead of focusing on segmenting the chromosomes based on histone codes or scanning for genes with differentially histone modification patterns, we integrated the data from temporal RNA-seq, temporal histone ChIP-seq, TF ChIP-seq assays in related cell types, and perturbation data, to systematically reconstruct the regulatory relationship in cardiac differentiation.

Compared with the original analysis of RNA-seq and histone ChIP-seq datasets by Wamstad et al. [1], the novelty of this study are two-fold: (1) instead of determining the TFBS by using a heuristic method to find the *dip* in the H3K27ac profile, followed by motif enrichment analysis, we used a logistic regression model that considered sequence, expression and histone modification features. This model computes the probability of identifying TF binding sites for any base in cis-regions, and quantitatively predicts the binding potentials of any TFs with known PWMs. This strategy allowed us to explore the binding profiles of a broader spectrum of TFs rather than the significantly enriched TFs. (2) In the network inference stage, instead of considering only the expression correlation, we used a time-varying dynamic Bayesian model that combines three different types of information including: (a) the LR score, (b) the temporal expression profiles and (c) experimentally derived perturbation data to quantitatively reconstruct the dynamic GRN during cardiac differentiation. This model is also able to predict the global outcome by computationally perturbing specific TFs.

The most significant advantage of our method over other network inference methods is that, by combining multi-dimensional data, it not only predicts the gene-gene relationship, but also pinpoints the specific TF binding sites in the cis-region. Our method successfully identified the known regulatory region (−9435 bp to −8922 bp) upstream of the transcriptional start site of the key cardiac gene *Nkx2-5*. In addition, graphical representation of the links illustrate the global landscape of the gene regulatory network and predicts novel factors whose function is yet to be discovered.

This method will allow biologists to quickly determine the potential *cis* modules that regulate important genes during cardiac differentiation and any biological processes that involve temporal cascade in gene induction, which can be experimentally tested in the laboratory. It also emphasizes the importance of analyzing the same system in multiple dimensions in a comparable manner.

Building upon our successful prediction of known gene-gene regulatory relationships and enhancers, there are multiple interesting ways that our current method can be extended. For example, microRNAs or long non-coding RNAs (lncRNAs) have been shown to play important roles in heart development [69,70]. Since their expression have also been profiled, for example, during the cardiac differentiation [1], it would be intriguing to integrate them into the current framework and build networks not only including transcription factors, but also microRNAs or lncRNAs [4,71]. To incorporate microRNA data, the development of strategies to measure the stage-specific binding relationship between a microRNA and a mRNA, rather than the 'static' relationship of a microRNA-mRNA pair predicted by most microRNA target prediction tools will be needed. By examining the 'static' microRNA target sites change on mRNAs under alternative polyadenylation is another way to incorporate microRNAs into the current framework. As for lncRNAs, we need a deeper understanding of the mechanism of how they regulate the target genes [72].

Another potential extension of our method will be an inter-specific comparison of gene regulatory mechanisms. For example, the temporal RNA-seq/histone ChIP-seq data have also been generated for human cardiac differentiation [3]. It will be interesting to systematically combine the human and mouse data to study the conserved regulatory network components or the network evolution [73]. The potential challenge is that these two studies used an overlapping, but different sets of histone states (H3K27ac, H3K4me3, H3K4me1, H3K27ac and RNAP [1], versus H3K27me3, H3K4me3, H3K36me3 and RNAP [3], and different time points for their analyses. Our model needs to be extended to accommodate datasets that have been collected in different experimental paradigms.

## Conclusion

In summary, we propose an integrative approach to utilize multi-dimensional gene expression, histone modification and transcriptional data. We predict that such a conceptual framework is crucial to fully decode the rapidly accumulating -omics data in the biological field.

## Methods

### Transcription factor ChIP-seq datasets

The ChIP-seq binding sites for 17 transcription factors in ESCs and HL-1 cells were downloaded from ChIPBase [2,26,38,52,74] (Table 1). The genomic coordinates were converted to the mouse genome version mm10. We used findMotifsGenome.pl in HOMER (v4.6) to identify the enriched motifs in each dataset with the default parameters [52]. The most significant PWM for each transcription factor was used to scan the *cis-region* of each gene to find the possible binding sites (*hit*) at either plus or minus strand with the PWM score of 90% of the highest possible score, by using *matchPWM()* from Biostrings package in Bioconductor. The *cis-region* is defined as 40 kb surrounding the transcription start site. If the cis-regions of neighboring genes were overlapped, the bases within overlapped regions were assigned to their nearest gene.

### Feature preparation and logistic regression model

The raw RNA-seq, histone modification (H3K4me3, H3K27me3, H3K4me1 and H3K27ac) and RNA polymerase II phosphorylation at serine 5 (RNAP) ChIP-seq data during cardiomyocyte differentiation from ESCs, mesoderm (MES), cardiac progenitors (CP) and cardiomyocytes (CM) were downloaded from NCBI GEO database (SRP026035 and SRP026036). The RNA-seq data were analyzed by TopHat (v2.0.11)/Cufflink (v2.1.1) pipeline [75]. The ChIP-seq reads were first mapped in the mouse genome mm10 by BWA (v0.7.4), followed by MACS (v1.4.1) analysis [76]. The 13,961 genes whose expression levels are greater than 1 FPKM in at least one of four time points were used for the following analysis. The ChIP-seq tag intensity for every 10 bp interval was transformed by an inverse hyperbolic sine function to reduce the distortion effects of high data values [77]. The features were calculated as described in Tables 2 and 3, followed by scaling to the mean of zero and a standard deviation of 1.0. The logistic regression models were trained by *glm()* function in R. The elastic net regularized logistic regression model was trained by *glmnet* package in R [78], while the complexity parameter *λ* was automatically determined using three fold cross-validation.

### Perturbation network

We compiled the perturbation experiments for 189 TFs in ESCs and 4 in HL-1 cells [2,4,20,50,51]. The significantly differentially expressed genes between control and induction (or repression) microarray samples were determined by RankProd [79] with FDR < 0.1. The perturbation network is represented as a 13,961 (number of expressed genes) by 13,961 matrix ***P*** where ***P***_*ij*_ = 1 if gene *i* is significantly up- or down-regulated after inducing or repressing gene *j,* otherwise, 0. There are 29,534 non-zero entities in the perturbation matrix ***P*** (Additional file 2: Table S3).

### Time-varying dynamic Bayesian network

Let ***X*** be a *p* by *T* expression level matrix, where *p* is the number of genes and *T* is the number of time points (log(FPKM + 1)). Let ***A***_*t*_ be a *p* by *p* coefficient matrix describing the regulatory relationship during the transition from *t* to *t + 1*, where *t* is from 1 to *T-1*. The dynamic expression levels can be modeled as

$$ {X}^{t+1}={A}^t{X}^t+\varepsilon,\;\varepsilon \in N\left(0,{\sigma}^2I\right) $$

based on time-varying dynamic Bayesian networks [13].

For each gene *i*, the regulatory relationship by other genes at the transition from *t* to *t + 1* can be further modeled as:

$$ {A}_i^t={\left({u}^{i,t}\right)}^T{B}^{i,t} $$

where ${\boldsymbol{A}}_{\boldsymbol{i.}}^{\boldsymbol{t}}$ is the row *i* of matrix ***A*** ^*t*^, ***u*** ^*i,t*^ is a *K* ^*i*^-length vector indicating the weight of *K* ^*i*^ cis-segments for gene *i*. In this study, the cis-segments were defined as 50 bp segments in the cis-region with the step size as 5 bp, that is, every adjacent segment have 45 bp overlap. ***B*** ^*i,t*^ is a *K* ^*i*^ by *p* binding profile matrix describing the binding potentials of each TF for each cis-segment *k*, and defined as:

$$ {\boldsymbol{B}}_{s(k),j}^{i,t}=\left\{\begin{array}{cc}\hfill {\displaystyle \sum_{h\in s(k)}\frac{\boldsymbol{L}{\boldsymbol{R}}_h^{i,t}}{\left|s(k)\right|}{\boldsymbol{\pi}}_{ij}}\hfill & \hfill \kern0.24em  if\; there\; exists\;a\;PWM\; hit\; of\;TF\;j\; in\;cis\mathit{\hbox{-}} segment\;k\hfill \\ {}\hfill 0\hfill & \hfill otherwise\hfill \end{array}\right. $$

where *s(k)* represents the location of cis-segment *k* with length |*s(k)*|, ***LR***_*h*_^*i,t*^ is the LR score of base *h* on cis-region of gene *i* at time *t*, and *s(k)* represents the bases contained within the cis-segment *k*. The parameter *π*_*ij*_ adjusts for the regulatory relationship existing in the corresponding perturbation experiments: if in the perturbation matrix, ***P***_*ij*_ = 1, then *π*_*ij*_ = 1, otherwise, *π*_*ij*_ = 0.25. At each time point *t*, only abundantly expressed TFs were used (FPKM > 25). There are 66, 70 and 61 abundantly expressed TFs (with known PWMs) in ESCs, MES and CPs (Additional file 1: Figure S4B). If two PWM hits were overlapped, the PWM hit with the higher score was used.

The network inference problem can be formulated as an optimization problem such that, for each gene *i*,

$$ \underset{u^{i,1},\dots, {u}^{i,T-1}}{min}\kern0.36em {\displaystyle \sum_{t-1}^{T-1}}{\left\Vert {X}_i^{t+1}-{\left({u}^{i,t}\right)}^T{B}^{i,t}{X}_i^t\right\Vert}_2+{\lambda}_1{\displaystyle \sum_{t-1}^{T-1}}{\left\Vert {u}^{i,t}\right\Vert}_1+{\lambda}_2{\displaystyle \sum_{t-1}^{T-2}}{\left\Vert {u}^{i,t}{B}^{i,t}-{u}^{i,t+1}{B}^{i,t+1}\right\Vert}_1 $$

The first term in the objective function is to minimize the difference between observed expression levels and expression levels that can be explained by the regulatory relationship. The second term is for obtaining a sparse weight of the cis-segments, that is, most of the cis-segments will have the weight of zero and only a few cis-segments have significant impact on the expression levels of nearby genes. The third term is to smooth the edge weights between the adjacent networks. Two hyperparameters *λ*_*1*_ and *λ*_*2*_ were selected by cross-validation. This optimization problem is convex for every gene and can be solved by standard convex optimization methods. In this study, we used the CVX convex modeling package (http://cvxr.com/cvx/).

To evaluate the confidence of each TFBS, we used a bootstrap method: for the cis-region of each gene, the LR scores were re-sampled with replacement, followed by network inference using the time-varying DBN. This process was repeated for *N* = 100 times for each gene. For each estimated ${\boldsymbol{u}}_{s(k)}^{i,t}$ (the weight of cis-segment *s(k)* of gene *i* at time *t*), the *p*-value was calculated as $\frac{{\displaystyle {\sum}_{n=1}^NH\left({}^nu>u\right)+1}}{N+1}$, where ^*n*^ *u* is the *n*-th bootstrapped estimation, *u* is the estimation without bootstrapping (the superscripts and subscripts were omitted to reduce the clutter), and *H(x)* = 1, if *x* > 0, otherwise 0 [80].

## Additional files

Additional file 1: Figure S1. (A-C) Performance of leave-one-TF-out cross-validation of predicting binding sites of 17 TFs, as measured by area under the ROC, with different parameters. (D) AUC of leave-one-TF-out cross-validation by using different models. **Figure S2** Distribution of a mean LR score of (A) ESC enhancers [36] and (B) weakly conserved heart enhancers [37] and a LR score of one million randomly selected bases in the cis-region. **Figure S3** (A) Comparing the feature coefficients estimated by the logistic regression model without regularization (glm), and with elastic net regularization (glmnet). (B-C) Comparing the LR score (chromosome 10, mm10) predicted by the logistic regression model without regularization (glm) and with LASSO regularization during three transitions. **Figure S4** Number of (A) expressed (FPKM > 1) and (B) abundantly expressed (FPKM > 25) transcription factors with known PWMs. **Figure S5** Predicted up- or down-regulated genes on computationally inducing (A) Tcf3, (B) Sox2, (C) Nanog, and (D) Zfp281 five-fold in ESCs, compared with known up- or down-regulated genes following the experimental induction of each corresponding gene. p-values were determined using Fisher's exact test. **Figure S6** Graphic representation of predicted TFBS for (A) Gata4, (B) Gata6 and (C) Bhlhe40. **Figure S7** An extended time-varying DBN that incorporates the effects of TF with unknown PWMs and non-TFs (group II genes). (A) Number of predicted outgoing edges for each group II gene in three transitions. (B) Number of group II genes with outgoing edges in three transitions. (C) The predicted sub-network that include 57 group I genes and 43 group II genes in CP-CM transitions.
Additional file 2: Table S1. Pathway analysis of TFs that are significantly enriched in high LR score regions in three transitions. **Table S2** The predicted regulatory relations of 13,961 expressed genes. **Table S3** The perturbation networks for 189 TFs in ESCs and 4 in HL-1 cells compiled from multiple studies.
Additional file 3:
**An extended time-varying dynamic Bayesian network (DBN) model for non-transcription factors and transcription factors with unknown positional weight matrices.**

## Abbreviations

ESC: Embryonic stem cells
MES: Mesoderm
CP: Cardiac progenitors
CM: Cardiomyocytes
TF: Transcription factors
GRN: Gene regulatory networks
TFBS: Transcription factor binding sites
PWM: Positional weight matrices
H3K4me1: Histone H3 Lys4 mono-methylation
H3K4me3: Histone H3 Lys4 tri-methylation
H3K27me3: Histone H3 Lys27 tri-methylation
H3K36me3: Histone H3 Lys36 tri-methylation
H3K27ac: Histone H3 Lys27 acetylation
RNAP: RNA polymerase II phosphorylation at serine 5
LOTFOCV: Leave-one-transcription-factor-out cross-validation
DBN: Dynamic Bayesian network
