# Supplementary material for: Inferring dynamic gene regulatory networks in cardiac differentiation through the integration of multi-dimensional data
Source: BMC Bioinformatics. 2015 Mar 7;16:74. doi: 10.1186/s12859-015-0460-0 (PMC4359553; doi:10.1186/s12859-015-0460-0)

Supplementary Figure 1

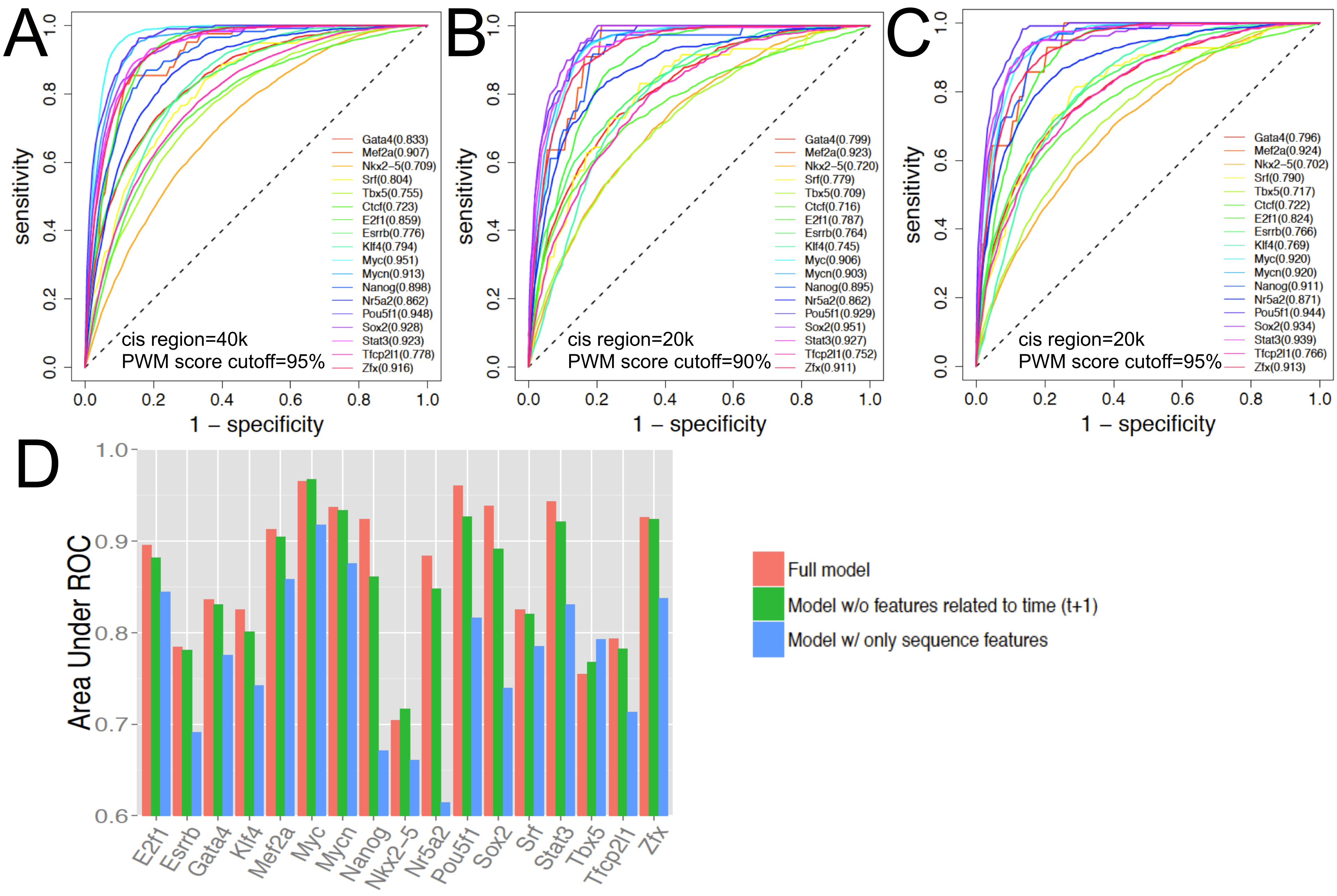

Supplementary Figure 2

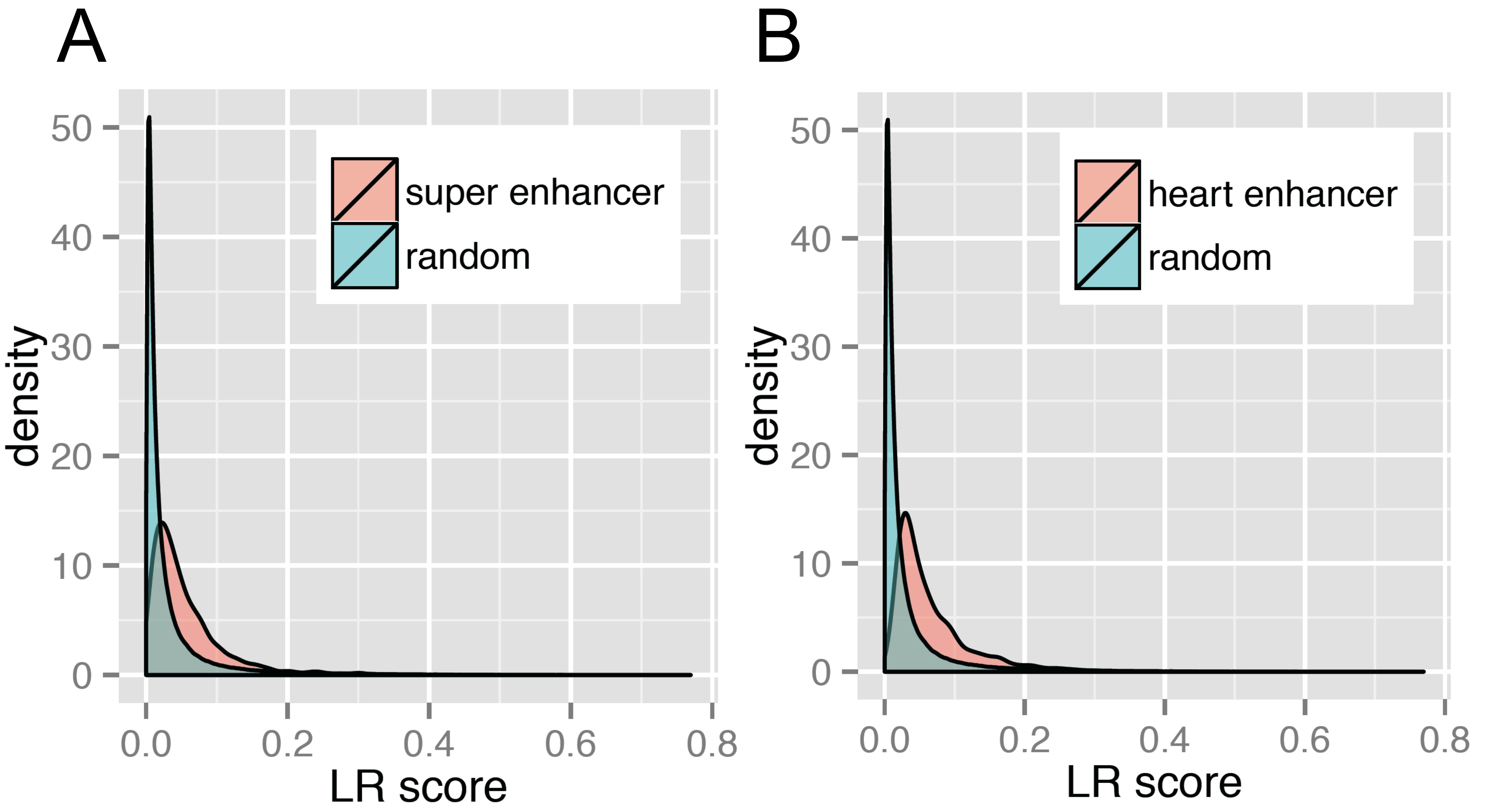

Supplementary Figure 3

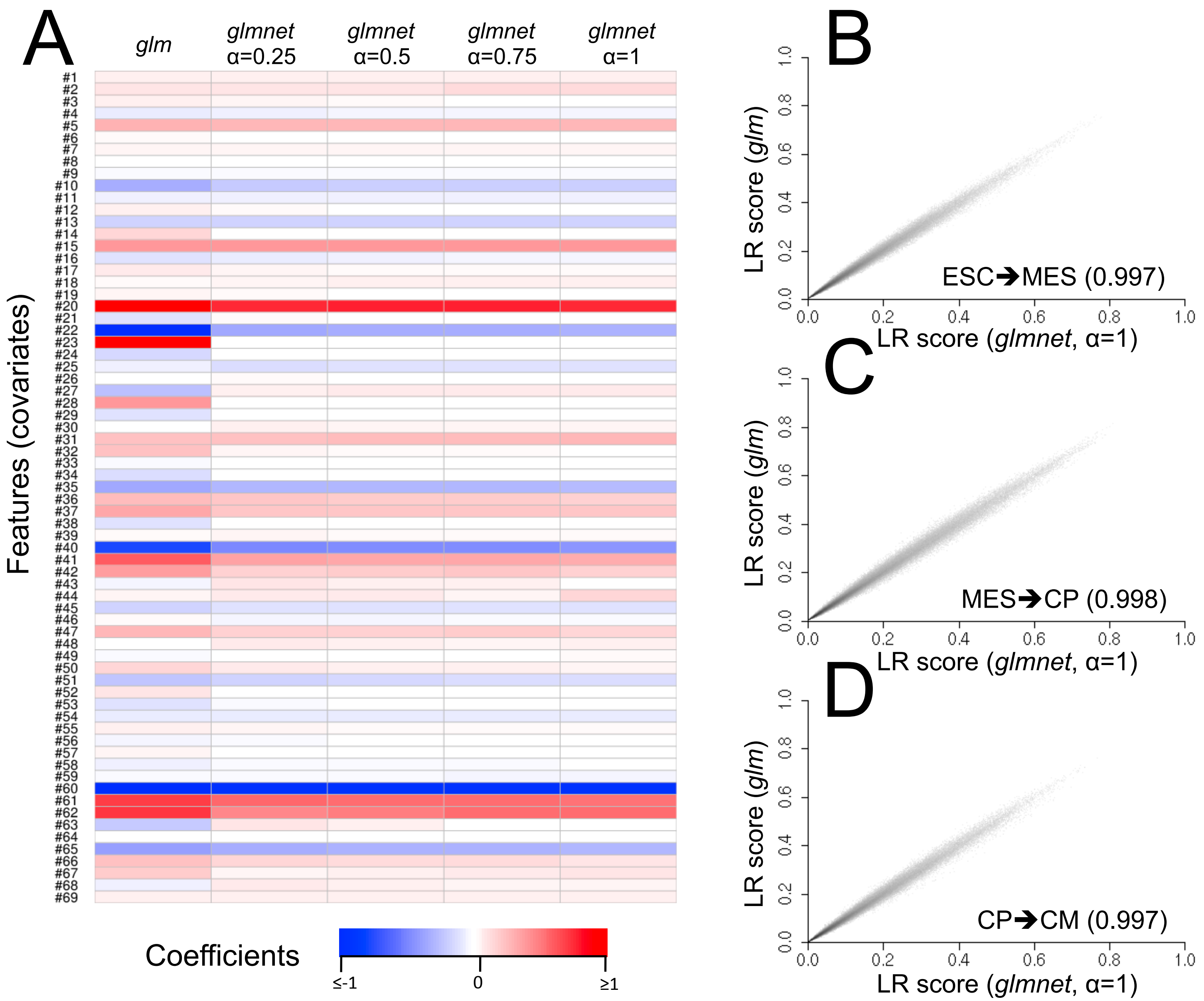

Supplementary Figure 4

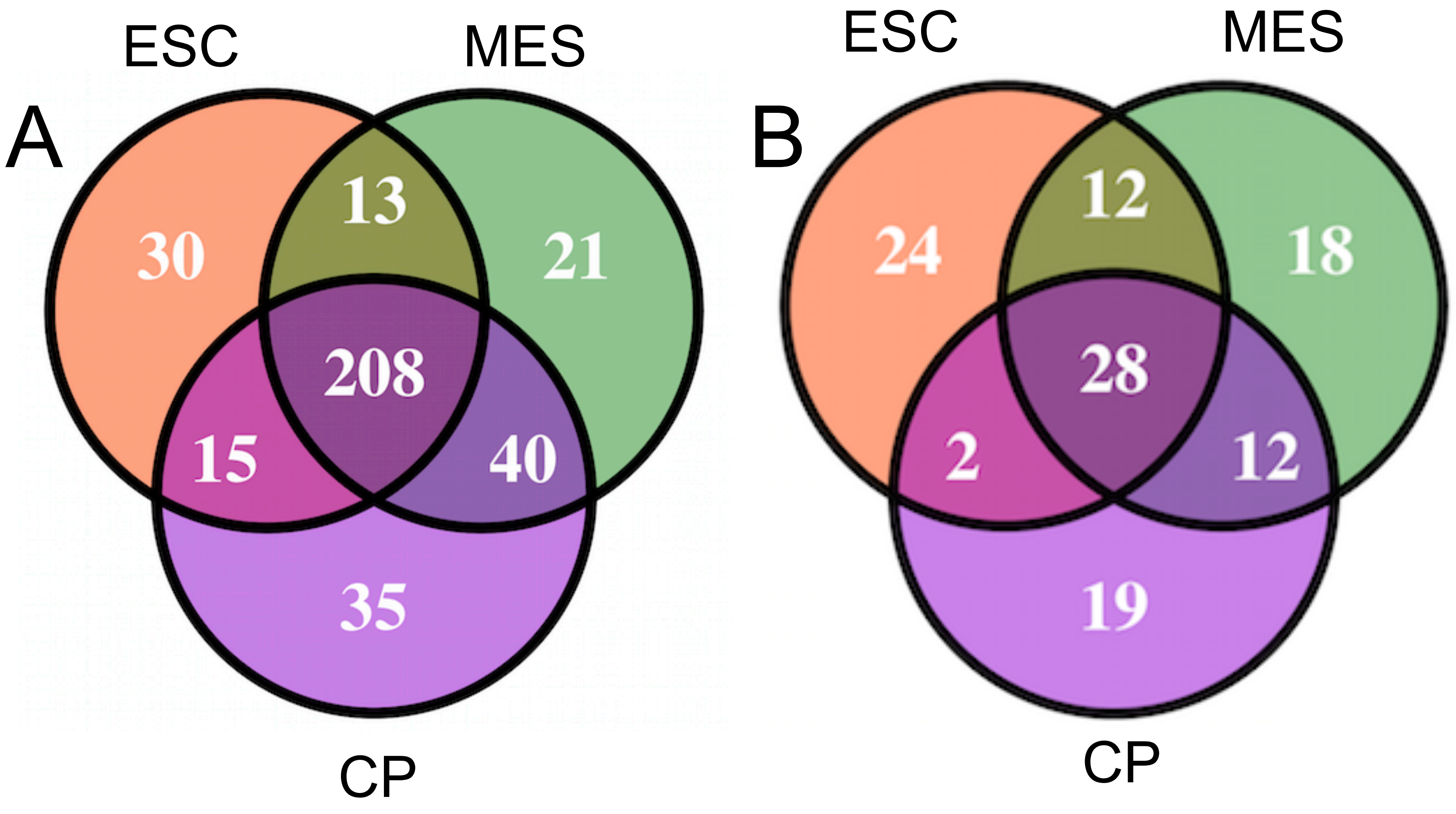

Supplementary Figure 5

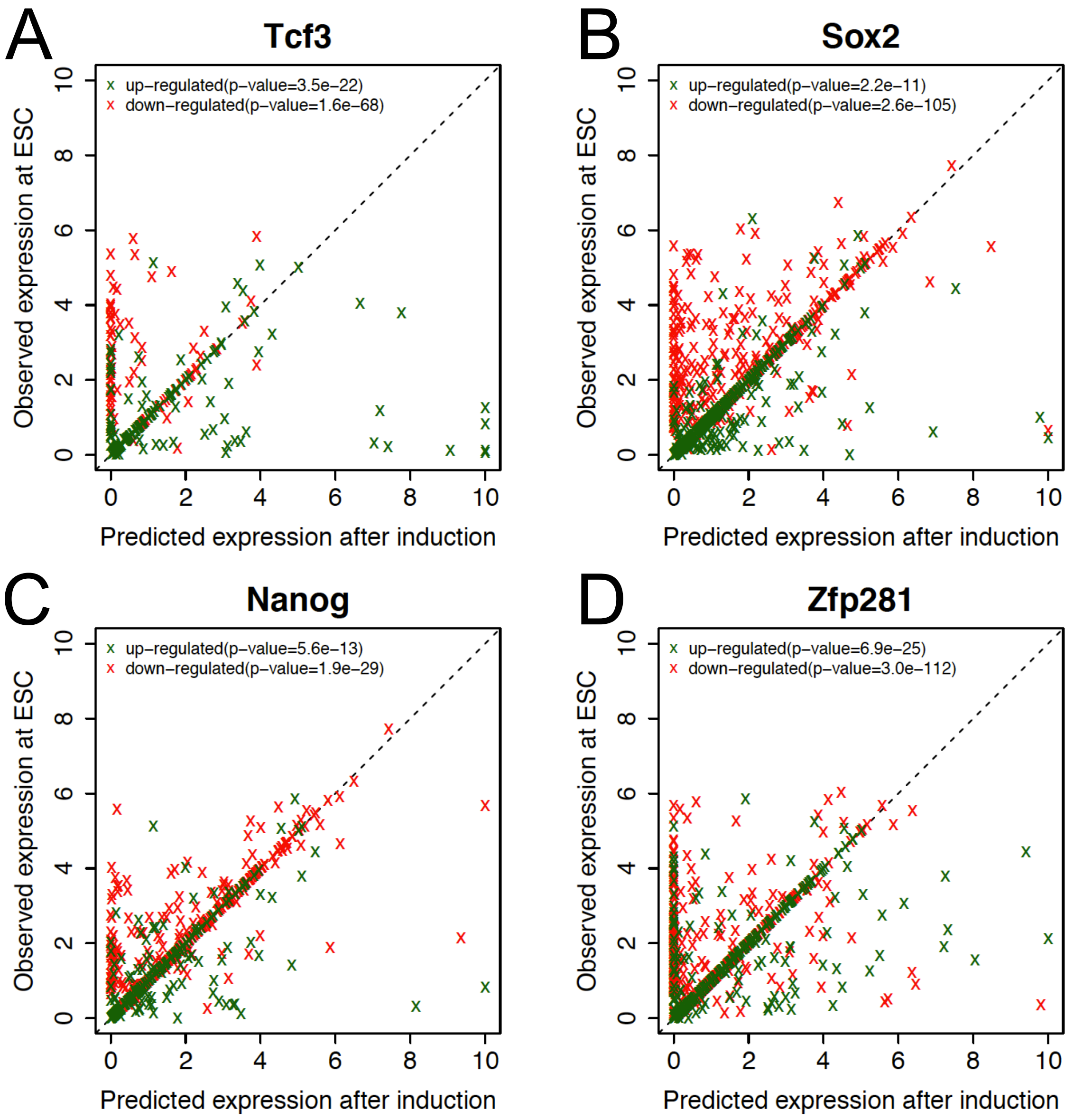

Supplementary Figure 6A

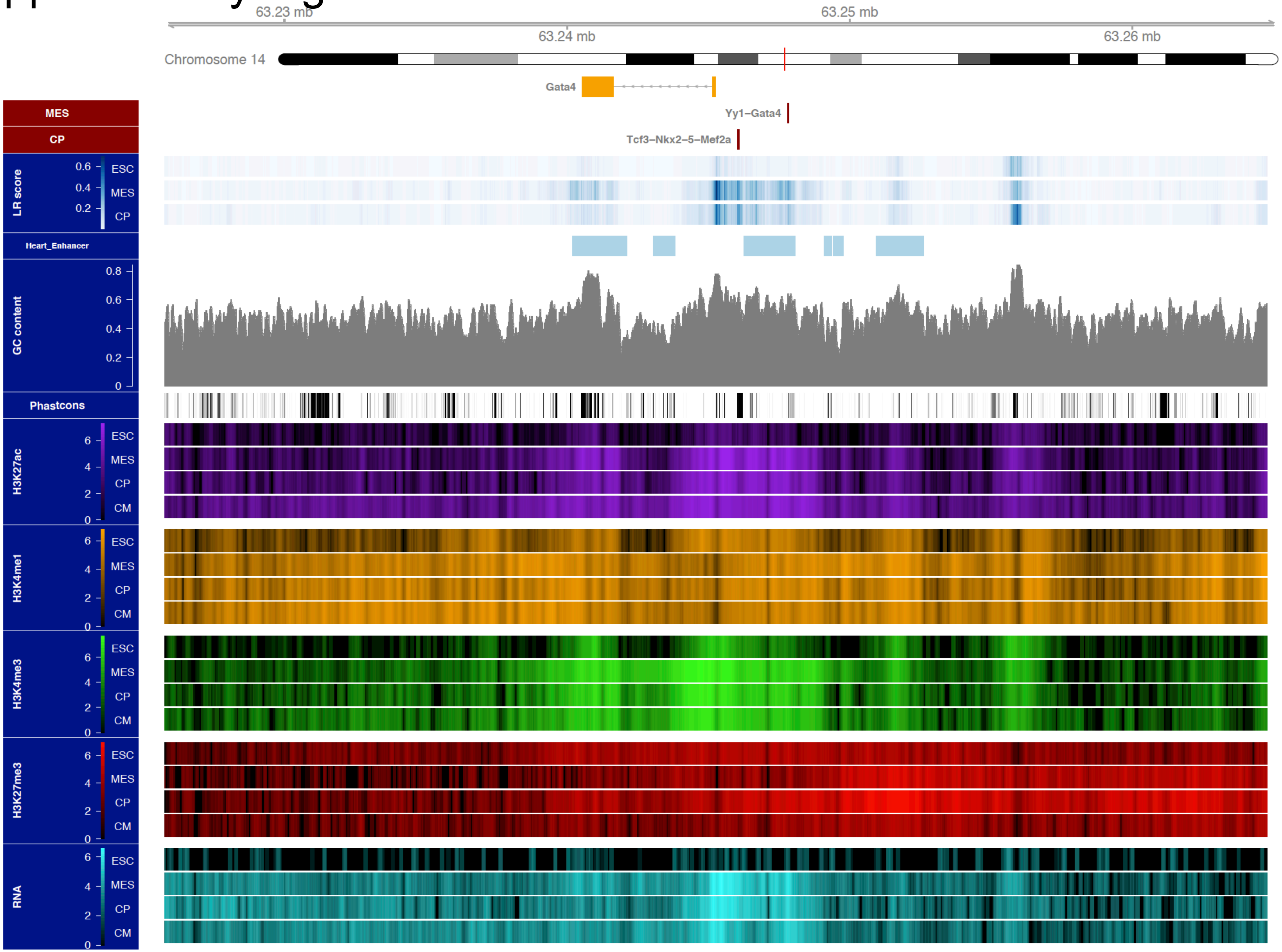

Supplementary Figure 6B

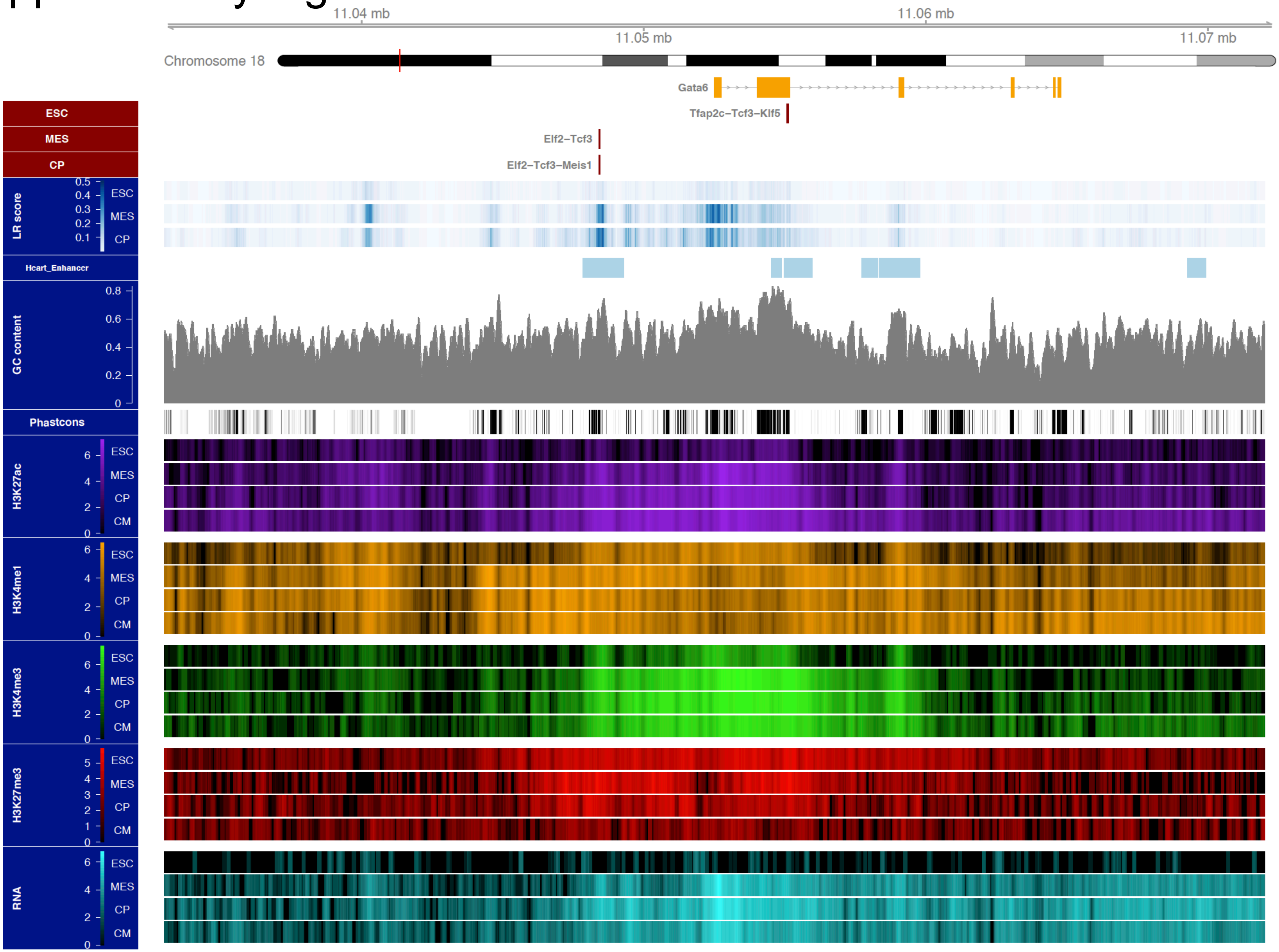

Supplementary Figure 6C

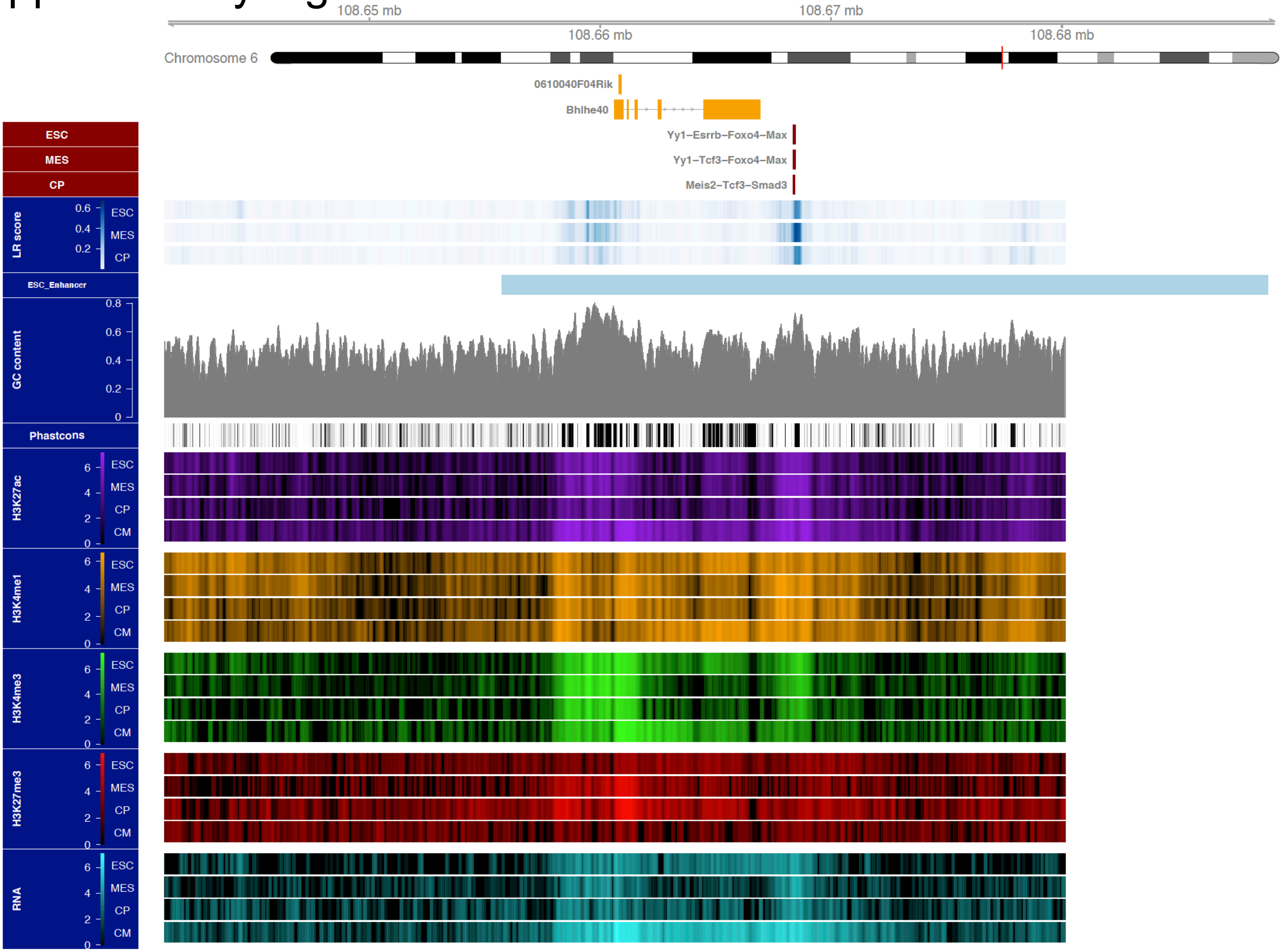

Supplementary Figure 7

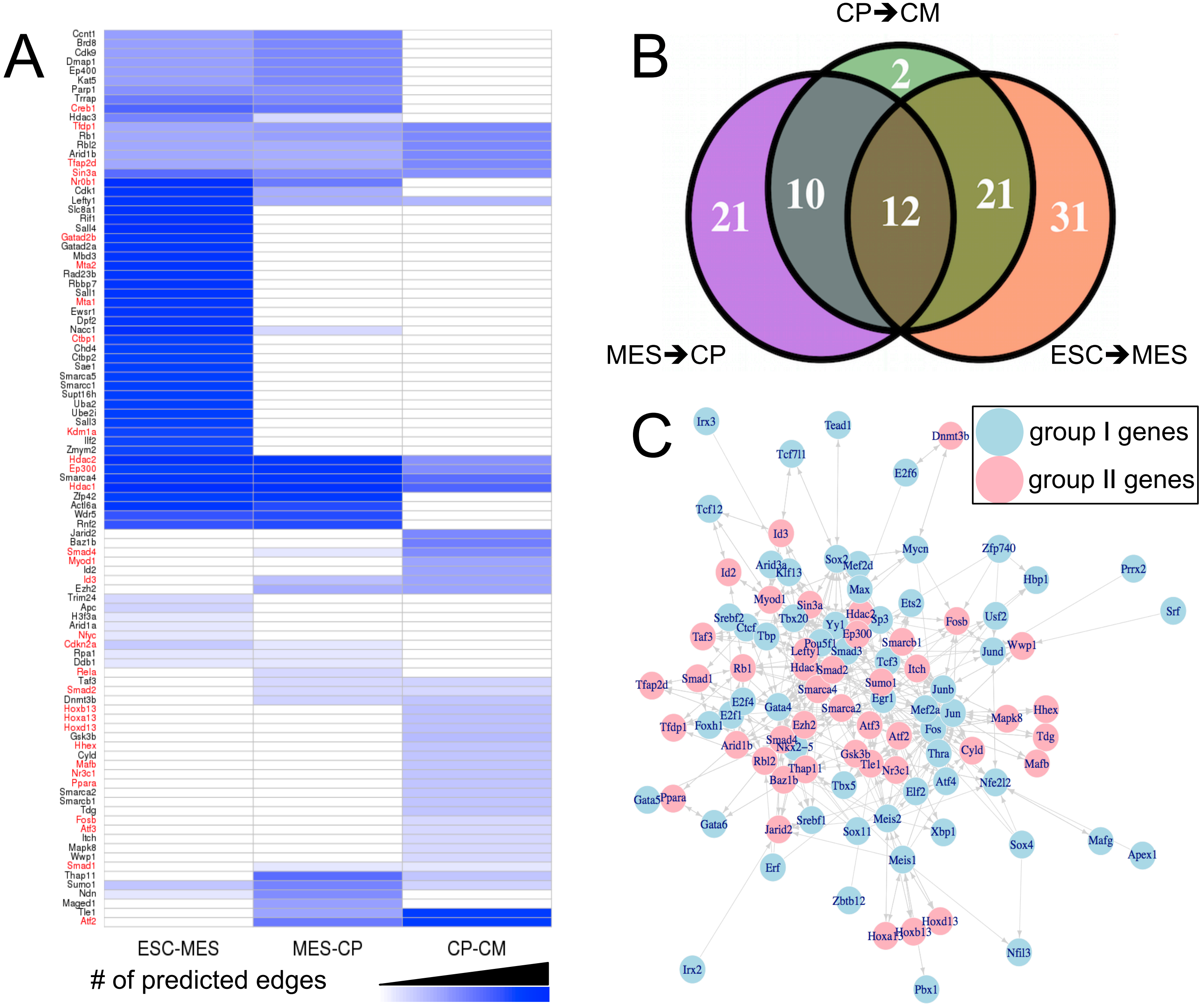

Supplement: Additional file 1: Figure S1. — (A-C) Performance of leave-one-TF-out cross-validation of predicting binding sites of 17 TFs, as measured by area under the ROC, with different parameters. (D) AUC of leave-one-TF-out cross-validation by using different models. Figure S2 Distribution of a mean LR score of (A) ESC enhancers [36] and (B) weakly conserved heart enhancers [37] and a LR score of one million randomly selected bases in the cis-region. Figure S3 (A) Comparing the feature coefficients estimated by the logistic regression model without regularization (glm), and with elastic net regularization (glmnet). (B-C) Comparing the LR score (chromosome 10, mm10) predicted by the logistic regression model without regularization (glm) and with LASSO regularization during three transitions. Figure S4 Number of (A) expressed (FPKM > 1) and (B) abundantly expressed (FPKM > 25) transcription factors with known PWMs. Figure S5 Predicted up- or down-regulated genes on computationally inducing (A) Tcf3, (B) Sox2, (C) Nanog, and (D) Zfp281 five-fold in ESCs, compared with known up- or down-regulated genes following the experimental induction of each corresponding gene. p-values were determined using Fisher's exact test. Figure S6 Graphic representation of predicted TFBS for (A) Gata4, (B) Gata6 and (C) Bhlhe40. Figure S7 An extended time-varying DBN that incorporates the effects of TF with unknown PWMs and non-TFs (group II genes). (A) Number of predicted outgoing edges for each group II gene in three transitions. (B) Number of group II genes with outgoing edges in three transitions. (C) The predicted sub-network that include 57 group I genes and 43 group II genes in CP-CM transitions. [file 12859_2015_460_MOESM1_ESM.pdf]
