# Supplementary material for: Inferring dynamic gene regulatory networks in cardiac differentiation through the integration of multi-dimensional data
Source: BMC Bioinformatics. 2015 Mar 7;16:74. doi: 10.1186/s12859-015-0460-0 (PMC4359553; doi:10.1186/s12859-015-0460-0)
Supplement: Additional file 3: — An extended time-varying dynamic Bayesian network (DBN) model for non-transcription factors and transcription factors with unknown positional weight matrices. [file 12859_2015_460_MOESM3_ESM.docx]

**An extended time-varying dynamic Bayesian network (DBN) model for non-transcription factors and transcription factors with unknown positional weight matrices**

Wuming Gong^1^, Naoko Koyano-Nakagawa^1^, Tongbin Li^2^  and Daniel J. Garry^1*^

^1^ Lillehei Heart Institute, University of Minnesota, 2231 6^th^ st. SE, 4-165 CCRB, Minneapolis, MN 55114, USA

^2^ AccuraScience LLC, 5721 Merle Hay Road, Suite #16B, Johnston, IA 50131, USA

*Corresponding Author

**Methods**

In the time-varying DBN we proposed in the main text, the vertices with outgoing edges are genes encoding transcription factors (TF) with known positional weight matrices (PWM) (*defined as group I genes*), which represent a small proportion of all known TFs (<30%). Moreover, non-DNA binding proteins, such as signaling proteins and components of the chromatin complex, also play important roles in ESC differentiation and heart development by collaborating with TFs [1-3]. We defined genes encoding the TFs with unknown PWMs and non-DNA binding proteins as the *group II genes*.

Here, we evaluate an extended model to incorporate the effect of group II genes into the time-varying DBN modeling. To model the regulatory effects of group II genes, we made an assumption that if an expressed (FPKM > 1) group II gene has an effect on the nearest gene, the protein of this group II genes must interact with the proteins of group I genes that have TFBS in at least one cis-segment in the cis-region.

We used the protein-protein interaction (PPI) data from STRING database (version 9.1) with 13,034 interactions with confidence score at least 0.4 (medium confidence or better) [4].

To incorporate the effects of group II genes, the binding profile matrix $\boldsymbol{B}_{s\left( k \right),j}^{i,t}$ for a group II gene *j* on cis-segment *s(k)* for nearby gene *i* at time *t* was defined as:

$$\boldsymbol{B}_{s\left( k \right),j}^{i,t}=\left\{ \begin{matrix} \sum_{p\in P_{s(k)}^{i,t}} \frac{\boldsymbol{B}_{s\left( k \right),p}^{i,t}}{|P_{s(k)}^{i,t}|} & if gene j interacts with all genes in set P_{s(k)}^{i,t} \\ 0 & otherwise \end{matrix} \right.$$

where $P_{s(k)}^{i,t}$ is a set of group I genes, whose binding profile is non-zero on cis-segment *s(k)* for nearby gene *i* at time *t*. In other words, if a group II gene *j* interacts with all group I genes that bind to a cis-segment of nearby gene *i*, the effect of gene *j* on nearby gene *i* is the mean effects of this set of group I genes.

By extending the model in this way, we incorporated the effects of group II genes, that is, non-TF genes or TF genes without known PWMs, into time-varying DBN.

**Results**

By using this extended model, we predicted 40,810, 7,814 and 2,335 additional gene-gene edges that involved 97 additional group II genes (Supplementary Figure 7A) in ESC-MES, MES-CP and CP-CM transitions, respectively. Among these group II genes, 37 genes were annotated with sequence-specific DNA binding transcription factor activity (GO:0003700) in Gene Ontology [5]. There were 64, 45 and 43 group II genes with outgoing edges in ESC-MES, MES-CP and CP-CM transitions, respectively (Supplementary Figure 7B). The most linked group II genes included Ep300, SWI/SNF complex members Actl6a, Smarca4, Mta2, Arid1b and Sall1, as well as histone deacetylase 1/2 (Hdac1 and Hdac2). It has been shown that SWI/SNF complex is required for pluripotency of mouse ESC, and deficiency of SWI/SNP components impaired the ability of mouse ESC to differentiate into three germ layers[3]. SWI/SNF complex is also required for cardiogenesis and regulates the function of key cardiac factors such as Nkx2-5 and Mef2c [1, 2].

The functional analysis of group II genes in three transitions suggested that mouse phenotypes such as abnormal heart morphology (MP:0000266), abnormal myocardium layer morphology (MP:0004056) and abnormal cardiac muscle tissue morphology (MP:0010630) are significantly enriched in 43 group II genes in the CP-CM transition (FDR < 1E-3) but not in the genes in ESC-MES transition [6]. The predicted sub-network that included 57 group I genes and 43 group II genes in the CP-CM transition are shown in Supplementary Figure 7C.

In summary, we provided an extension of our original model with the capability of incorporating the effects of group II genes into the time-varying DBN modeling. The results suggested that this extended model successfully predicted the additional gene regulatory pathways (such as SWI/SNF complex) that are important for heart development. However, this extended model relies on the quality of the PPI data, which are still largely incomplete and static. We believe that, although demonstrated to be feasible, further improvement of the modeling process awaits dynamic and more accurate PPI data.

**References**

1. Lei I, Gao X, Sham MH, Wang Z: **SWI/SNF protein component BAF250a regulates cardiac progenitor cell differentiation by modulating chromatin accessibility during second heart field development.** *Journal of Biological Chemistry* 2012, **287**:24255–24262.

2. Lickert H, Takeuchi JK, Both Von I, Walls JR, McAuliffe F, Adamson SL, Henkelman RM, Wrana JL, Rossant J, Bruneau BG: **Baf60c is essential for function of BAF chromatin remodelling complexes in heart development.** *Nature* 2004, **432**:107–112.

3. Yan Z, Wang Z, Sharova L, Sharov AA, Ling C, Piao Y, Aiba K, Matoba R, Wang W, Ko MSH: **BAF250B-associated SWI/SNF chromatin-remodeling complex is required to maintain undifferentiated mouse embryonic stem cells.** *Stem Cells* 2008, **26**:1155–1165.

4. Franceschini A, Szklarczyk D, Frankild S, Kuhn M, Simonovic M, Roth A, Lin J, Minguez P, Bork P, Mering von C, Jensen LJ: **STRING v9.1: protein-protein interaction networks, with increased coverage and integration.** *Nucleic Acids Res* 2013, **41**(Database issue):D808–15.

5. Gene Ontology Consortium, Blake JA, Dolan M, Drabkin H, Hill DP, Li N, Sitnikov D, Bridges S, Burgess S, Buza T, McCarthy F, Peddinti D, Pillai L, Carbon S, Dietze H, Ireland A, Lewis SE, Mungall CJ, Gaudet P, Chrisholm RL, Fey P, Kibbe WA, Basu S, Siegele DA, McIntosh BK, Renfro DP, Zweifel AE, Hu JC, Brown NH, Tweedie S, et al.: **Gene Ontology annotations and resources.** *Nucleic Acids Res* 2013, **41**(Database issue):D530–5.

6. Chen J, Bardes EE, Aronow BJ, Jegga AG: **ToppGene Suite for gene list enrichment analysis and candidate gene prioritization.** *Nucleic Acids Res* 2009, **37**(Web Server issue):W305–11.
